# Sound Insulation Properties of Hollow Polystyrene Spheres/Polyethylene Glycol/Epoxy Composites

**DOI:** 10.3390/polym14071388

**Published:** 2022-03-29

**Authors:** Xuejun Shi, Guangling Shi, Songtian Li, Xiangxiang Du, Yongjun Han

**Affiliations:** College of Chemistry and Chemical Engineering, Pingdingshan University, Pingdingshan 467000, China; 2782@pdsu.edu.cn (X.S.); 2780@pdsu.edu.cn (G.S.); 2702@pdsu.edu.cn (S.L.)

**Keywords:** epoxy resin, hollow polystyrene spheres, composites, sound transmission loss

## Abstract

The generation of noise requires a noise source, transmission path, and passive acceptance target of noise, all of which are indispensable. Blocking the propagation path of noise is one of the available means when the existence of the noise source and passive receiving target cannot be addressed. This is an effective way to prevent noise pollution, often using sound insulation materials to block the path of noise transmission. In this work, composites with excellent sound insulation properties were designed and prepared. These composites, using epoxy resin (EP) as the matrix, polyethylene glycol (PEG), and hollow polystyrene spheres (HPS), were added to epoxy resin as a toughening agent and functional filler to prepare the ternary HPS/PEG/EP composites. The soundproofing results showed that when the thickness of the sample was 3 mm, the average sound transmission loss (STL) value of the neat EP and the HPS/PEG/EP composites with an HPS 32 vol% was up to 19.0 dB and 42.1 dB, and the STL values of the composites were increased by approximately 120% compared to the pure epoxy. When the sample was 10 mm thick, the average STL value of the HPS/PEG/EP composites with HPS 32 vol% contents was enhanced to 55.7 dB.

## 1. Introduction 

Noise pollution, such as traffic noise, construction noise, industrial noise, and society noise, is the by-product of modern industrial and economic development in the world. These noises induce sleep disturbances, a lack of psychosocial well-being, annoyance, hearing loss, hypertension, and psychiatric disorders [1,2,3]. Methods to easily eliminate the noise problem have become more and more important for people who frequently endure this noise torture. Of course, controlling noise sources is a very effective way to eliminate the noise problem, but it is impossible to control all noise sources because people cannot stop using all machines at any one time [4]. Therefore, people have had to take other approaches to solve noise pollution. So, insulation materials have been used to block the sound wave diffusion path, and this has been proven to be an effective method by many researchers. 

Researchers have invented many kinds of insulation materials to decrease noise pollution [5,6,7], such as single-layer insulation materials [8], double-layer insulation materials [9,10], and sandwich-structure insulation materials [11,12,13]. Among these insulation materials, sandwich-structure insulation materials constitute an important direction for the development of sound insulation materials, of which the middle interlayer of the sandwich materials can be composed of piezoelectric plate materials [14], phenolic foam [15], polyurethane foam materials [16], honeycomb acoustic material [17], silica aerogels [18], and so on. Therefore, this novel structure material has attracted the attention of many researchers. However, these sandwich-layer structure materials bring certain disadvantages: Unstable interfaces, high fabrication costs, and a complex production process. Therefore, easily enhancing single-layer materials’ soundproofing ability through simple methods became a research hotspot that attracted more and more researchers’ attention, and this strategy was much more meaningful because the multilayer-structure sound insulation materials and sandwich-structure insulation materials could be prepared from single materials. Fortunately, scholars conducted a great deal of research in this area and many useful studies can now be used for reference.

Wang investigated the sound insulation effect of mica/PVC composites and the experimental results show that the STL value of mica/PVC was perceptibly improved due to the addition of the mica [19]. Ahmadi investigated the sound insulation properties of clay-filled acrylonitrile–butadiene–styrene (ABS) composites and found that ABS/clay (4 wt% and 8 wt%) could improve the sound transmission loss (STL) of pure ABS by 15 and 5 dB at high- and low-frequency ranges, respectively [20]. Kim and Kang studied the soundproofing properties of PP/Clay/carbon nanotube (CNT) composites and found that the STL for the PP/4.8 wt% clay/0.5 wt% CNT composite was approximately 20 dB higher than that of pure PP at high frequencies and approximately 12 dB higher at low frequencies. They also assessed PP/CNT/xGnP composites and found the STL of the 80/10/10 wt% PP/CNT/xGnP composite to be more than 5 dB higher than the pure PP [21,22]. Liang studied the sound insulation properties of glass-bead-filled PVC/glass ball (GB) and hollow-glass-bead-filled PP/hollow glass ball (HGB) composites and found that the soundproofing property of the PP/HGB composite was higher than the PVC/GB composite [23,24]. 

These results proved that hollow fillers (CNT, HGB) and inorganic particles (Mica, Clay) could improve the sound insulation and mechanical properties of the composites. Our group also conducted research wherein hollow silica nanotubes (HSNTs) were synthesized by the sol-gel method, and the HSNTs were used as functional fillers to fabricate a novel sound insulation composite (epoxy/HSNTs) based on epoxy resin (EP), and the EP/HSNTs composites showed excellent performances [25]. On the basis of this work, epoxy resin was used as the matrix, hollow polystyrene spheres were used as functional fillers, polyethylene glycol was used as a toughening agent and a thickener, and the ternary HPS/PEG/EP composites were prepared using blending technology. This design strategy was expected to obtain sufficient sound insulation properties as a result of the HPS, as well as excellent mechanical properties from the PEG for use in the ternary composites [26,27]. 

## 2. Experiment Part

### 2.1. Materials

Bisphenol A epoxy resin (epoxy, E-44, EP), with an epoxy value of 0.41~0.47(EQ/100 g), a density of 1180 kg/m^3^, and purity ≥ 95%, was bought from Shanghai Resin Factory Company (Shanghai, China). Polyethylene glycol (PEG), with an average relative molecular weight of 600 and a density of approximately 1270 kg/m^3^, was purchased from Beijing Sinopharm Chemical Co., Ltd., Beijing, China. The hollow polystyrene spheres (HPSs), with a particle size of approximately 600–750 nm, a density of 126 kg/m^3^, an outer diameter of approximately 600–700 nm, an inner diameter of approximately 450–550 nm, and a shell thickness of approximately 150 nm, was presented by the research group of Beijing University of chemical technology. The curing agent, namely methyl hexahydrophthalic anhydride (MeHHPA), with a density of 1.162 g/mL and purity ≥ 97%, was purchased from Zhejiang Jiaxing Qingyang Chemical Co., Ltd., Jiaxing, China. The accelerator, namely 2-ethyl-4-methylimidazole (EMI-2,4), with a density of 0.975 g/mL and a purity ≥ 96%, was purchased from Hubei Wuhan Yuancheng Gong chuang Technology Co., Wuhan, China. Other common reagents were purchased from Beijing Chemical Reagent Co., Beijing, China.

### 2.2. Preparation of Composites

A certain amount of HPS and PEG were mixed directly with the epoxy resin using a Rotation–Revolution Hybrid Mixer (ARE-310, THINKY, Tokyo, Japan). The MeHHPA and EMI-2 and 4 were added to the above mixture at room temperature. After undergoing mixing for 15 min and defoaming for 3 min, the resin mixture was poured into the pre-heated steel molds coated with the release agent and cured at 60 °C for 4 h and 100 °C for 6 h. In this research, the weight ratio of the PEG was 10 wt% for the total quality of epoxy and MeHHPA, and the additive amount of HPS was represented by the volume fraction for the resin mixture.

The bending test results showed that the PEG/EP composites have excellent bending properties, and the experimental results are shown in Table 1. The bending properties of the PEG/EP composites were first enhanced and then decreased as the PEG content increased, and when the PEG loading was 10 wt%, the bending results of the PEG/EP composites were best, a result also proven by Siamak Zavareh in his article [26]. In this recipe design, the HPS and matrix were not layered. In this research, the PEG content was selected to be 10 wt% in the PEG/EP and HPS/PEG/EP composites. Two types of composites, namely HPS/PEG/EP and HPS/EP, were prepared in this research, and their soundproofing properties were contrastively discussed.

### 2.3. Measurements and Characterization

**Figure 1:**
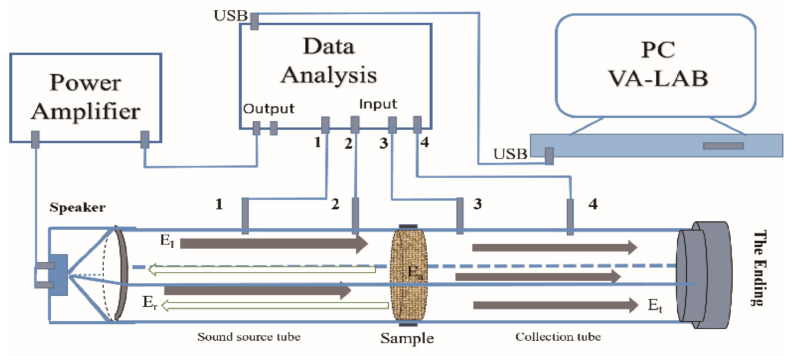
Scheme of impedance tube for measuring STL with four microphones.

The STL values were measured at room temperature by the impedance tube (Figure 1). The test setup (Beijing Prestige Acoustic Technology Co., Ltd, Beijing, China) consisted of four microphones, a sound calibrator, a BSWA-SW466 type impedance tube, a computer with VA-LAB, a conditioning amplifier, and a frequency analyzer. The STL test values were in accordance with the method in the ASTM E2611 standard.

Transmission electron microscopy (TEM) images were obtained on a Tecnai G220 electron microscope (FEI Co., Amsterdam, The Netherlands) with an acceleration voltage of 200 kV. Samples were prepared by placing a drop of the diluted ethanol-suspended HPS on copper grids and drying it under ambient conditions. The morphology and size of the HPS and the dispersions on the composite fracture surfaces were studied with a field emission scanning electron microscope (FE-SEM, FEI Co., Amsterdam, Netherlands) at an accelerating voltage of 10 kV. HPS samples for SEM studies were also prepared by placing a drop of the diluted ethanol-suspended HPS on a mica sheet and drying it under ambient conditions. All samples were sputtered with platinum prior to SEM examination.

Dynamic mechanical analysis (DMA) of the composites was performed by the TA Q800. The tests were carried out using the single cantilever mode, and the specimen dimensions were 35 × 10.0 × 4 mm^3^. The heating rate was 5 °C/min, the frequency was 1 Hz, the amplitude was 25 μm, and the temperature range was 30–210 °C.

Electronic universal test machines (Shenzhen San Testing Machine co., CMT4104, Shenzhen, China) were used to test the bending properties of the composites. Additionally, the bending test samples were prepared according to the ISO 178: 2010 standard. Each reported value is a result of the average of at least 6 specimens.

### 2.4. Theoretical Basis of Sound Transmission Loss

The scheme of the impedance tubes is shown in Figure 1. Sound energy was produced by the speaker in the sound source tube, the sample was located in the middle of the tube, and four microphones were placed on their stations. The microphones measured the sound power, converted it to a signal, and input the signal into the computer. This signal was calculated and changed to an STL value by the computer. The results of the STL values were calculated by Equation (1) using the transfer function method.

STL = *−*20lg|E_t_/E_I_| (1)

In this experiment, “E_t_” represents the transmitting sound energy, “E_I_” is the incident sound energy, “E_r_” is the reflecting sound energy, and “E_a_” is the absorbing sound energy in Figure 1. The relationship of these factors was as follows: E_I_ = E_t_ + E_r_ + E. Whether the incident sound energy was reflected or absorbed by the samples, the incident sound energy was eliminated and weakened. 

**Figure 2:**
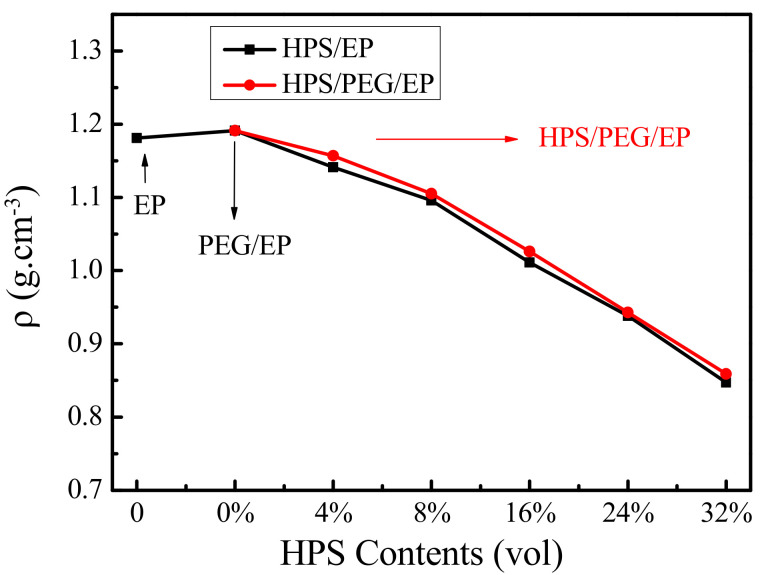
The densities of the pure EP, PEG/EP, HPS/EP and HPS/PEG/EP composites.

The density of the composite can affect the sound insulation properties, and according to the mass law, under the same conditions, the heavier the density of the material, the higher the STL values [28,29]. As the density test results showed, the density of the PEG/EP sample was higher than that of pure EP and the density of the HPS/PEG/EP sample was higher than the HPS/EP composites, as shown in Figure 2. This figure also showed that the density of all the composites decreased with an increase in the HPS loading, which decreased and approached 0.85 g/cm^3^ when the HPS content was 32 vol%. Therefore, we were required to consider the density change of the composites in this experiment, as the content of the HPS was higher, the densities of the composites were lower, and lower densities were not conducive to the sound insulation performance of the composites.

## 3. Results and Discussion

### 3.1. Dispersion of Hollow Polystyrene Spheres in the PEG and Epoxy Matrix

**Figure 3:**
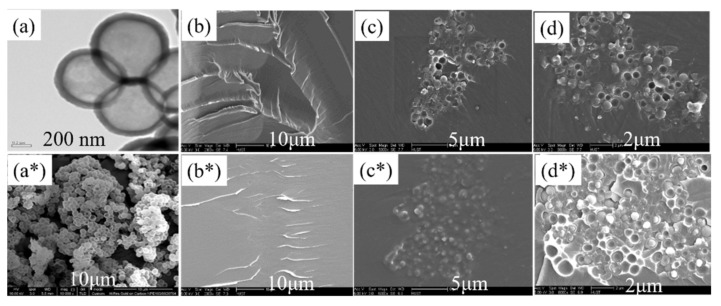
(**a**) TEM image of the HPS, (a*) SEM image of the HPS, (**b**,b*) SEM images of the fracture surface of EP and PEG/EP, (**c**,c*) SEM images of the HPS/EP and HPS/PEG/EP composites with the same HPS loading of 8 vol%, (**d**,d*) SEM images of the HPS/EP and HPS/PEG/EP composites with the same HPS loading of 24 vol%.

TEM and SEM were used to examine the morphology of hollow polystyrene spheres, and SEM was also used to observe the fracture surface of the composites. As shown in Figure 3a,a*, most hollow polystyrene spheres were empty, and the inner diameter was approximately 600 nm, the outer diameter was approximately 750 nm, and the shell thickness was approximately 150 nm. We can also observe that the interior of the HPS was empty, which lays the foundation for the sound insulation performance of the composite. The fracture surfaces of the EP matrix and PEG/EP composites are shown in Figure 3b,b*, and the river morphology of the PEG/EP composites was more exquisite than the pure EP matrix.

The dispersion of the HPS in the matrix was investigated using SEM from the fracture surface of the composites. Figure 3c,c* shows that the loading of HPS was 4 vol% in the two typical HPS/EP and HPS/PEG/EP composites, and Figure 3c* shows the HPS/PEG/EP composite’s fracture surface. From these images, we can determine that the fracture surface of the HPS/PEG/EP composites was fuzzy, which was due to the use of PEG as a toughening agent in the matrix. In Figure 3d,d*, the loading of HPS is 24 vol%, and the figure also shows the HPS/PEG/EP composite’s fracture surface. From these images, we can observe that part of the HPS embedded in the matrix and the other part of the HPS left traces of holes in the fracture surface. It can also be seen from the SEM image that residual parts of some HPSs following crushing are embedded in the epoxy resin matrix, and the interior of these broken HPS are empty. Therefore, this indicates that the HPS builds many small spaces in the composite, and most of the interior space of the HPS is preserved during the curing process of the epoxy resin. This was because the surface of the HPS had many carboxyl groups, and these groups enhanced the force between the HPS and epoxy matrix. At the same time, the HPS formed slight aggregation, which became more and more obvious as the HPS content increased.

### 3.2. Dynamic Mechanical Properties of the Composites

**Figure 4:**
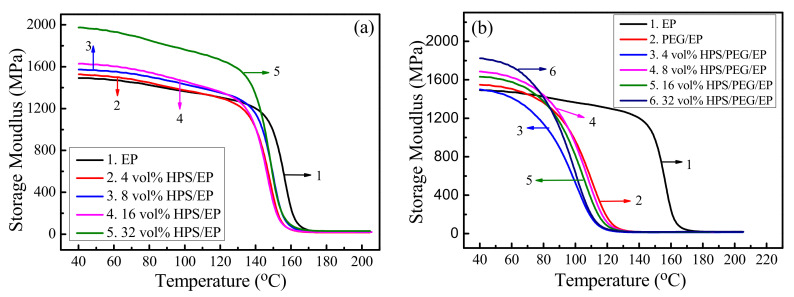
(**a**) The storage modulus results of EP and HPS/EP composites; (**b**) the storage modulus of EP and HPS/PEG/EP composites.

Dynamic mechanical tests were performed to investigate the physical and chemical structural changes of the polymers and composites over a wide range of temperatures [30]. Figure 4 shows the curves of the storage modulus (E′) versus temperature, and, more specifically, Figure 4b shows the storage modulus of the HPS/PEG/EP composites. As shown in Figure 4a, compared with the neat epoxy, the storage modulus of the HPS/EP composite became higher and higher as the HPS loading increased. In Figure 4b, we can also observe that when the temperature was below 80 °C, the storage modulus of the HPS/PEG/EP composite was higher than the neat EP and EP/HPS composites, but when the temperature was above 90 °C, the storage modulus of the HPS/PEG/EP composite decreased very quickly and the values were all lower than the neat epoxy. This is because the 10 wt% PEG molecular chain inserted into the epoxy matrix caused the epoxy molecular chain to become softer, and therefore it slid more easily in the high-temperature region.

**Figure 5:**
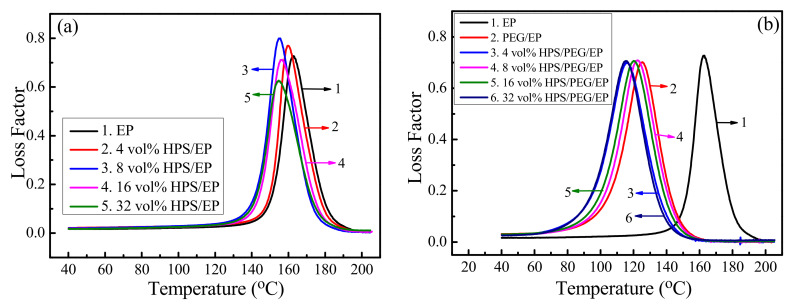
(**a**) The loss factors of the EP and HPS/EP composites; (**b**) the loss factors of the EP and HPS/PEG/EP composites.

The loss factor versus temperature is given in Figure 5, and Figure 5b shows the loss factor of the HPS/PEG/EP composites. Upon comparing Figure 5a,b, we can clearly observe that the T_g_ values of the HPS/PEG/EP and HPS/EP composites were all lower than the neat epoxy. In Figure 5b, this trend is very clear, and the T_g_ values of HPS/PEG/EP composites became lower and lower and finally reached 110 °C. The maximum values of the loss factor barely changed, but the half peak width became wider. This was because the molecular chains of PEG were inserted into the epoxy matrix and thus lowered the inner stress of the epoxy matrix chains and promoted the moving space of the epoxy polymer chains, and these molecular chains could then absorb more sound energy [27,31,32].

### 3.3. Sound Insulation Properties of the Composites

**Figure 6:**
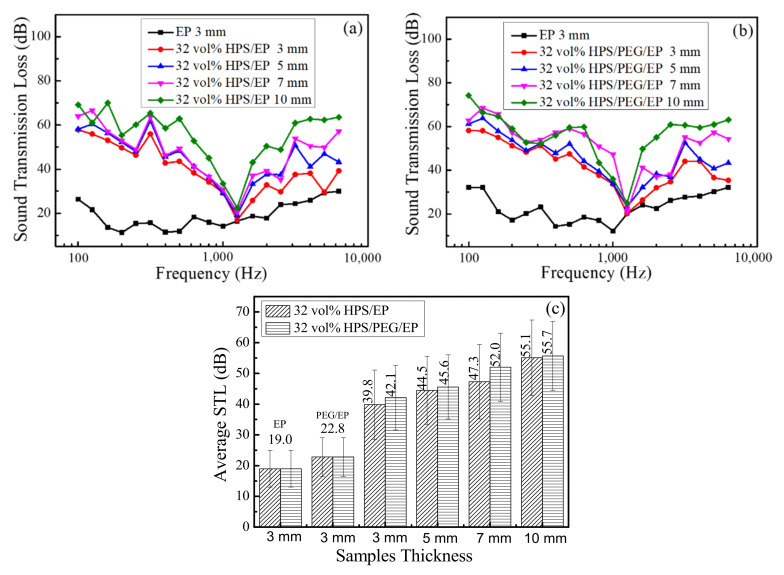
(**a**) Soundproof properties of the EP and HPS/EP composites with different thicknesses; (**b**) soundproof properties of the EP and HPS/PEG/EP composites with different thicknesses; (**c**) the average STL of the EP, HPS/PEG/EP, and the HPS/PEG/EP composites with different thicknesses.

The STL values were tested at 100 Hz to 6300 Hz and the average STL values were obtained from one-third octave average values [25,33]. The soundproofing properties and average STL values of the HPS/EP and HPS/PEG/EP composites with different thicknesses are shown in Figure 6a–c. The loadings of HPS remained at 32 vol% and the thickness of the samples ranged from 3 mm to 10 mm. The STL values of the HPS/EP and HPS/PEG/EP composites increased with the increasing thickness of the samples across all frequencies. The average STL values of the neat epoxy, PEG/EP, HPS/EP, and HPS/PEG/EP composites with 3 mm thickness were 19 dB, 22.8 dB, 39.8 dB, and 42.1 dB, respectively. The average STL values of the HPS/EP and the HPS/PEG/EP composites with 10 mm thickness were up to 55.1 dB and 55.7 dB, respectively. This phenomenon may be explained by the fact that, by mass law, the STL values relied on the thickness of the composites, and when the thickness of the material was higher, the STL value was greater.

When the thickness of the composites was set at a certain value, the functional hollow filler, such as the HPS, would play an important role in improving the STL value of composites, because the space of the HPS acted as the sound energy insulation barrier to weaken the sound wave.

**Figure 7:**
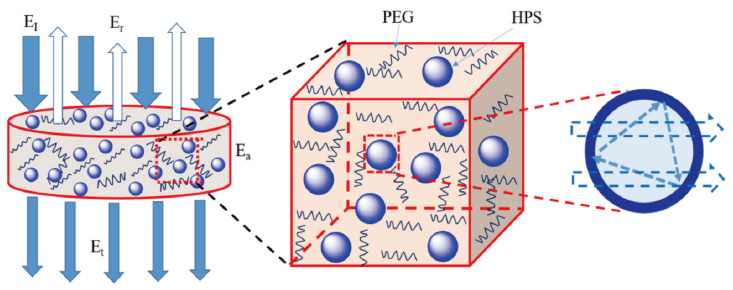
Scheme of the soundwave dispersion into the HPS/PEG/EP composites.

Figure 7 shows the scheme of the sound wave disperses into the composite materials. When the incident sound energy (E_I_) faced the smooth surface of the composite material, some sound energy was reflected (E_r_) and did not enter the composite, other incident sound energies (E_I_) transformed into mechanical vibration and entered the composite, and the remaining energy passed through the composite. The energy that passed through the composite was called the transmitting sound energy (E_t_), while the energy that was absorbed was named the absorption of sound energy (E_a_). In the HPS/PEG/EP composites, this sound energy gradually decreased. Because the matrix of the epoxy has damping properties similar to other polymers, the movement of polymer chains absorbed some energy and changed it to heat energy; in the meantime, the chains of the PEG also absorbed part of the energy, which were expended by the matrix. Secondly, the pathway of the mechanical vibration was extended. The interfaces between the HPS and epoxy matrix and the interfaces between the HPS and the air caused the soundwave to scatter, diffract, and refract; this process is accurately shown in Figure 7. Thirdly, the air inside the HPS space was confined in a narrow space and the air could move freely, akin to sound barriers, and the sound energy was decreased and weakened.

**Figure 8:**
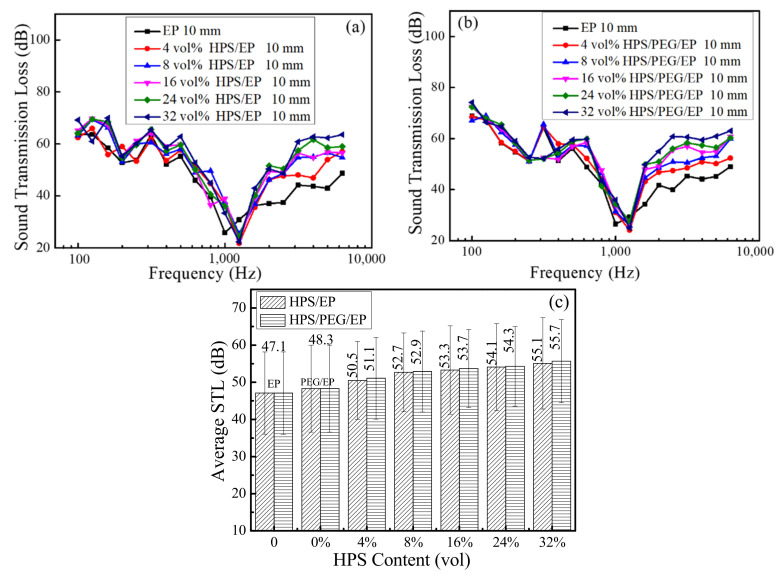
(**a**) Soundproof properties of the EP and the HPS/EP composites with different content of the HPS; (**b**) soundproof properties of the EP and the HPS/PEG/EP composites with different content of the HPS; (**c**) the average STL of the EP, HPS/PEG/EP, and the HPS/PEG/EP composites with different content of the HPS.

In Figure 8, all the sample thicknesses were 10 mm and the HPS loading ranged from 4 vol% to 32 vol%. The PEG loading in the HPS/PEG/EP composites was 10 wt%. The STL value of the HPS/PEG/EP and HPS/EP composites all increased as the HPS contents increased, across all frequencies. The average STL values of the neat epoxy, PEG/EP, HPS/EP, and HPS/PEG/EP composites with a 4 vol% HPS content reached up to 47.1 dB, 48.3 dB, 50.5 dB, and 51.1 dB, respectively. When the HPS content increased to 8 vol%, the average STL values of the HPS/EP and the HPS/PEG/EP composites increased up to 52.7 dB and 52.9 dB. Moreover, when the HPS loading was 32 vol%, the average STL values of the HPS/EP and the HPS/PEG/EP composites were up to 55.1 dB and 55.7 dB. At the same thickness of the composites, the densities of the PEG/EP, HPS/EP, and HPS/PEG/EP composites decreased as the HPS loading increased, and the lower density of the composites had a significant weakening effect on the sound insulation for the materials. From the above soundproofing values of the composites, we can more clearly determine the sound insulation potential function of hollow polystyrene spheres for the composites.

**Figure 9:**
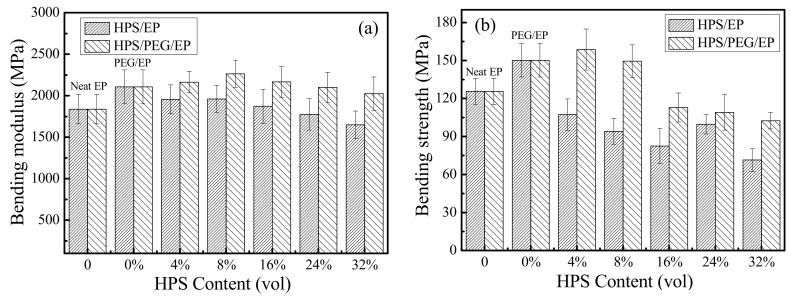
(**a**) The bending modulus of the EP, PEG/EP, HPS/EP, and HPS/PEG/EP composites; (**b**) the bending strength of the EP, PEG/EP, HPS/EP, and HPS/PEG/EP composites.

The excellent mechanical properties of the composite are the basic conditions for maintaining its sound insulation performance and other functions [34,35,36,37]. Therefore, we tested the bending performance of the EP, HPS/EP, and HPS/PEG/EP composites, and the test results are shown in Figure 9. It was found that there was a slight increase in the bending modulus of all the composites with the addition of HPS, as shown in Figure 9a. The bending strength was also enhanced with the increased loading of HPS in the composites, and the bending strength of the PEG/EP composites was higher than the neat epoxy, as shown in Figure 9b. When the content of the HPS was 4 vol%, the bending strength of the HPS/PEG/EP composites was up to 158.7 MPa, compared to the bending strength of the neat epoxy, showing an approximately 34.0 MPa improvement. The PEG chains embedded into the epoxy matrix may have decreased the internal stress of the epoxy matrix, and therefore the bending strength of the PEG/EP and the HPS/PEG/EP composites was improved. Furthermore, when the content of the HPS was higher than 4 vol%, the bending strength of the HPS/PEG/EP composites decreased.

## 4. Conclusions

In this work, ternary HPS/PEG/EP composites were designed and prepared. The sound insulation results proved that the ternary composites have excellent sound insulation performances, mainly because the cavity and interface of the hollow microspheres greatly prolonged the propagation path of soundwaves in the composites. The sound insulation results show that this is an effective strategy to improve the sound insulation performance of composites via adding hollow polystyrene microspheres. At the same time, by adding flexible segment material to the epoxy resin, the acoustic damping and bending performance of the composite can be enhanced. This work will provide new ideas and materials for research on sound insulation, as well as a new scheme to solve the problem of noise pollution.

## Figures and Tables

**Table 1:** Bending properties of the EP and PEG/EP composites with different PEG contents.

Sample	PEG Content (wt%)	Bending Strength (MPa)	Bending Modulus (MPa)
Neat EP	0	125.6 ± 10.4	1838 ± 176
PEG/EP-3%	3	130.3 ± 15.8	1897 ± 191
PEG/EP-7%	7	146.7 ± 16.3	1953 ± 189
PEG/EP-10%	10	150.2 ± 13.7	2106 ± 204
PEG/EP-15%	15	137.5 ± 18.1	1910 ± 180

## Data Availability

The data presented in this study are available on request from the corresponding author.
